# Functionalized synchrotron in-line phase-contrast computed tomography: a novel approach for simultaneous quantification of structural alterations and localization of barium-labelled alveolar macrophages within mouse lung samples

**DOI:** 10.1107/S1600577514021730

**Published:** 2015-01-01

**Authors:** Christian Dullin, Simeone dal Monego, Emanuel Larsson, Sara Mohammadi, Martin Krenkel, Chiara Garrovo, Stefania Biffi, Andrea Lorenzon, Andrea Markus, Joanna Napp, Tim Salditt, Agostino Accardo, Frauke Alves, Giuliana Tromba

**Affiliations:** a Institute for Diagnostic and Interventional Radiology, University Medical Center Göttingen, Robert Koch Strasse 40, 37075 Göttingen, Germany; b Cluster in Biomedicine, AREA Science Park Basovizza, Trieste, Italy; c Elettra Sincrotrone Trieste, Strada Statale 14, km 163.5 in AREA Science Park, 34149 Basovizza (Trieste), Italy; d Department of Architecture and Engineering, University of Trieste, Trieste, Italy; e Department of Physics, Chemistry and Biology, Linköping University, SE-581 83 Linkoeping, Sweden; f Institute for X-ray Physics, University of Göttingen, Göttingen, Germany; g Institute for Maternal and Child Health, IRCCS Burlo Garofolo, Trieste, Italy; h Department of Haematology and Medical Oncology, University Medical Center Göttingen, Robert Koch Strasse 40, 37075 Göttingen, Germany; i Molecular Biology of Neuronal Signals, Max Planck Institute for Experimental Medicine, Hermann-Rein-Strasse 3, 37075 Göttingen, Germany

**Keywords:** phase-contrast CT, single-distance phase retrieval, functional CT imaging

## Abstract

Functionalized computed tomography (CT) in combination with labelled cells is virtually non-existent due to the limited sensitivity of X-ray-absorption-based imaging, but would be highly desirable to realise cell tracking studies in entire organisms. In this study we applied in-line free propagation X-ray phase-contrast CT (XPCT) in an allergic asthma mouse model to assess structural changes as well as the biodistribution of barium-labelled macrophages in lung tissue. Alveolar macrophages that were barium-sulfate-loaded and fluorescent-labelled were instilled intratracheally into asthmatic and control mice. Mice were sacrificed after 24 h, lungs were kept *in situ*, inflated with air and scanned utilizing XPCT at the SYRMEP beamline (Elettra Synchrotron Light Source, Italy). Single-distance phase retrieval was used to generate data sets with ten times greater contrast-to-noise ratio than absorption-based CT (in our setup), thus allowing to depict and quantify structural hallmarks of asthmatic lungs such as reduced air volume, obstruction of airways and increased soft-tissue content. Furthermore, we found a higher concentration as well as a specific accumulation of the barium-labelled macrophages in asthmatic lung tissue. It is believe that XPCT will be beneficial in preclinical asthma research for both the assessment of therapeutic response as well as the analysis of the role of the recruitment of macrophages to inflammatory sites.

## Abstract 2

This study presents an approach to increase the sensitivity of lung computed tomography (CT) imaging by utilizing in-line phase contrast CT in combination with single-distance phase-retrieval algorithms and a dedicated image-processing regime. As demonstrated here, functional CT imaging can be achieved for the assessment of both structural alterations in asthmatic mouse lung tissue and the accumulation pattern of instilled barium-sulfate-labelled macrophages in comparison with healthy controls.

## 1. Introduction   

Lung imaging, especially of the mouse, remains extremely challenging due to the small size and high porosity nature of the organ, which creates various problems like scattering for X-ray and optical imaging techniques, susceptibility artefacts in magnetic resonance imaging (MRI) and shadowing of medical ultrasound waves at the rib cage. Together with preclinical airway disease models, novel imaging technologies are becoming increasingly important for monitoring disease progression and the efficacy of treatment within the lung. Recent studies have either focused on functional aspects, utilizing near-infrared fluorescence (NIRF) imaging (Napp *et al.*, 2010 ▶; Markus *et al.*, 2014 ▶) or on the depiction of morphological alterations, such as airway wall thickening using *ex vivo* micro-CT (Cortez-Retamozo *et al.*, 2008 ▶; Sera *et al.*, 2007 ▶). For tracking of *ex vivo* labelled immune cells *in vivo* a wide range of imaging modalities, for example MRI (Ahrens & Bulte, 2013 ▶) and optical imaging (Bousso & Moreau, 2012 ▶), have been reported in recent years. Radionuclide labelling of cells is the oldest technique for tracking immune cells, especially in whole body distribution studies in humans (Thakur, 1977 ▶). The application of CT in combination with novel contrast agents for cell tracking has just begun to be explored and is hampered by the poor sensitivity of CT combined with the low toleration of high concentrations of contrast media loaded into cells (Cormode *et al.*, 2014 ▶). In order to visualize the biodistribution of labelled cells following injection, as well as to assess morphological alterations in great detail during inflammation and airway remodelling within the lung, the combination of high spatial resolution with increased sensitivity is highly desirable. An imaging technique that meets these requirements is the here-used in-line free-propagation X-ray phase contrast CT (XPCT).

Since the very first application of X-rays for medical purposes by Konrad Roentgen in 1895, the imaging principle for detecting tissue-dependent variations by X-ray absorption has remained unchanged. Especially in applications related to visualization of soft-tissue as in mammography or in lung imaging, these differences in X-ray absorption are very weak, resulting in poor contrast. This contrast could be raised by lowering the X-ray photon energy; however, this would also increase the radiation dose deposition in the samples. Additionally, an energy level high enough to achieve sufficient penetration of the sample needs to be maintained. Due to these factors the absorption contrast in radiographs is limited, especially in clinical practice. In addition to absorption, a phase shift of the incident X-ray wavefront occurs within the sample. Despite being about 100 times stronger in low-density materials (Takeda *et al.*, 1995 ▶), this effect has basically not been exploited in clinical routine to date, due to the fact that at least partial coherent X-rays are required, which can only be generated with micro-focus X-ray tubes, or with higher intensities, at synchrotron light sources (Nugent, 2010 ▶). This limitation notwithstanding, X-ray phase-contrast imaging has great potential, as it combines strong edge enhancement in radiographs with the fact that the advantage of phase contrast over conventional absorption contrast improves with increasing photon energy. Therefore, medical phase-contrast X-ray imaging could potentially be performed at higher energies than in the actual absorption-based regime, which would reduce the dose deposition within the patient, especially in soft-tissue applications like mammography. There are already some clinical mammography systems on the market demonstrating a gain in image contrast due to phase effects (Tanaka *et al.*, 2005 ▶). Besides the here-used in-line free-propagation XPCT, there are other phase-sensitive techniques such as grating interferometry (Pfeiffer *et al.*, 2006 ▶). Grating interferometry would allow phase-contrast imaging even when conventional X-ray tubes are used. Notwithstanding that the clinical application of this technique is at the moment hampered by technical problems such as the magnitude of the applied radiation dose, grating interferometry also showed very promising results in lung imaging as demonstrated by Schleede *et al.* (2012 ▶).

Since absorption-based CT imaging in its nature shows low sensitivity, functional imaging approaches similar to those used in SPECT, PET or optical imaging (Nahrendorf *et al.*, 2008 ▶) are virtually nonexistent. The potential of phase-sensitive techniques for lung imaging has been explored since the late 1990s at some synchrotron beamlines (Yagi *et al.*, 1999 ▶; Kitchen *et al.*, 2004 ▶; Lewis *et al.*, 2005 ▶; Hooper *et al.*, 2007 ▶, 2009 ▶). However, the increased sensitivity of XPCT in less dense materials might be well suited to realise CT functional imaging addressing both soft-tissue alterations and the distribution of heavy-ion-based contrast agents such as barium sulfate. Contrary to classical absorption-based CT, in XPCT edge enhancement caused by phase effects at tissue interfaces is superimposed on the contrast caused by tissue-specific differences in X-ray absorption. These interactions of X-rays with matter are described by the complex refractive index 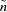 = 1 − δ + *i*β, where δ determines the phase shift and β the absorption. In order to analyse the phase shift separately, the δ part of the signal needs to be decoupled by a phase-retrieval (PhR) algorithm. It has already been demonstrated that the application of PhR is highly beneficial in the analysis of biological samples (Keyriläinen *et al.*, 2010 ▶; Zhang *et al.*, 2011 ▶; Yong *et al.*, 2009 ▶; Gureyev *et al.*, 2013 ▶) showing an up to 200-fold improvement in the contrast-to-noise ratio (Beltran *et al.*, 2011 ▶). A large variety of PhR algorithms have been reported so far, that can be loosely divided into multi- and single-distance techniques (Nugent, 2007 ▶).

Commonly used multi-distance PhR approaches are either based on the fact that the absorption part of the detected radiograph is constant whereas the impact of the phase effects varies with different sample-to-detector distances, or require *a priori* knowledge of the refractive indexes within the sample (Cloetens *et al.*, 1999*a* ▶; Beltran *et al.*, 2010 ▶). Imaging with several sample-to-detector distances is disadvantageous because it crucially depends on a perfect alignment of the different scans and it is affected by variations in the X-ray beam, which often occur at synchrotron light sources. In contrast to that, single-distance PhR algorithms minimize the scanning time and are therefore especially suitable for the analysis of unfixed biological samples such as *in situ* mouse lungs imaged in this study, but they can strictly only be applied on objects expressing a constant δ-to-β ratio (Paganin *et al.*, 2002 ▶). However, the applicability of this type of algorithm for soft-tissue samples was shown by Wu *et al.* (2005 ▶). Moreover, single-distance PhR followed by standard filtered backprojection reconstruction (FBP) was applied to generate three-dimensional (3D) data sets of mouse lungs predominately presenting the distribution of the δ part of the refractive index (Mohammadi *et al.*, 2014 ▶).

The aim of this study was to develop a functional XPCT imaging approach (fXPCT) by exploiting the capability of alveolar macrophages (MΦ) to migrate to inflammatory sites within the lung using an ovalbumin induced experimental allergic airways disease model (Markus *et al.*, 2014 ▶). In order to visualize the biodistribution of cells following intratracheal application, MΦ of the immortalized alveolar macrophage cell line MH-S (Mbawuike & Herscowitz, 1989 ▶) were *ex vivo* labelled by adding a contrast agent suitable for X-ray-based imaging directly to the cell culture media. A barium sulfate suspension was used, that is commonly applied in the clinic to mark the gastrointestinal tract in CT (Golder *et al.*, 1991 ▶). Thereby, macrophages were intracellularly labelled and used as a specific probe instead of an *in situ* labelling approach by systemic administration of X-ray contrast agents. MΦ can engulf large particles by phagocytosis and can therefore easily be loaded with contrast agents and drugs *in vitro* (Trivedi *et al.*, 2006 ▶), converting them into potential carriers for both diagnostic and therapeutic agents. Since they migrate to inflammatory sites they have already been exploited for delivery of various anti-inflammatory compounds (Bang *et al.*, 2011 ▶; Moreira & Hogaboam, 2011 ▶; Yang *et al.*, 2012 ▶). Moreover, MΦ were recently identified as one of the main effector cells in asthma (Bang *et al.*, 2011 ▶; Moreira & Hogaboam, 2011 ▶; Yang *et al.*, 2012 ▶). Mizue *et al.* (2005 ▶) showed that, in the absence of the macrophage migration inhibitory factor (MIF), asthma could not be induced in MIF-deficient mice, and Chen *et al.* (2010*a* ▶) demonstrated in an asthma mouse model that airway remodelling was successfully inhibited by a MIF antagonist.

With the unique capabilities of XPCT in combination with phase retrieval and the use of MΦ loaded with barium sulfate particles, we were able to simultaneously depict and quantify structural features and to illustrate in 3D the different accumulation sites of labelled MΦ within asthmatic and control lungs. We believe that, by providing new quantitative functional and anatomical parameters and by using barium-labelled immune cells in cell trafficking studies, this novel fXPCT approach may help to preclinically investigate complex and multi-factorial processes of inflammatory diseases.

## 2. Material and methods   

### 2.1. Preparation and labelling of macrophages   

The immortalized mouse alveolar MΦ cell line MH-S (purchased from American Type Culture Collection, ATCC, USA) was maintained in RPMI medium, supplemented with 10% FCS and 0.05 m*M* 2-mercaptoethanol (Mbawuike & Herscowitz, 1989 ▶) in a humidified atmosphere at 5% CO_2_ and 310 K. For CT imaging, cells were loaded with a barium sulfate suspension, the clinically used contrast agent Micropaque CT (Guerbet, France) by co-incubating 1 × 10^6^ cells ml^−1^ for 24 h with 3.5 µl Micropaque CT/ml cell media (175 µg barium sulfate/ml media), followed by two washing steps with phosphate buffer (PBS). Subsequently, for stable fluorescent labelling of the cell membrane, the MΦ were incubated for 30 min with 5 µl ml^−1^ of the lipophilic dialkylcarbocyanine dye Vibrant DiD (DiD; Molecular Probes, Eugene, OR, USA; excitation maximum: 644 nm; emission maximum: 665 nm), followed by two washing steps with PBS. To test the loading efficacy of barium sulfate particles into MΦ, a vial containing 1 × 10^5^ barium sulfate MΦ resuspended in 100 µl PBS was scanned with a normal bench-top microCT (eXplore locus SP, GE HealthCare, USA) (Verdelis *et al.*, 2011 ▶). The morphology of the barium-labelled MΦ was assessed by light microscopy and the effects of the uptake of barium sulfate particles on the metabolic activity of MΦ were investigated with a water-soluble tetrazolium (WST-1) cell proliferation assay (Madison, WI, USA) (Mosmann, 1983 ▶).

### 2.2. Mouse model of allergic asthma   

Female BALB/c mice (4–6 weeks old) were purchased from Harlan Laboratories and maintained with ‘*ad libitum*’ food and water. For generation of the experimental allergic airways disease model, mice were sensitized intraperitoneally (i.p.) at day 0 and 21 with 10 µg ovalbumin (OVA), dissolved in 200 µl PBS. At day 28 and 29, mice were treated intranasally (i.n.) with a solution of 100 µg OVA/50 µl PBS to induce an acute allergic reaction (Biffi *et al.*, 2013 ▶). Healthy age and gender-matched BALB/c mice, immunized and challenged with PBS only, were used as controls. To verify the success of OVA immunization, blood samples were taken 72 h after the last challenge from the facial vein of the living mice and levels of OVA-specific immunoglobulin within the sera were analysed as previously described (Biffi *et al.*, 2013 ▶).

Animal *in vivo* procedures were performed at the CBM Animal Facility, Trieste, Italy, in compliance with the guidelines of the European (86/609/EEC), the Italian (DL116/92) and at the University Medical Center Göttingen, Germany, in accordance with the German ethical laws (33.9-42502-04-10/0134) and were approved by the Italian Ministry of Health as well as by the animal ethics administration of Lower Saxony, Germany.

### 2.3. Application of macrophages and experimental setup   

Previous differential cell counts from bronchoalveolar lavages (BAL) and *in vivo* fluorescence measurement experiments showed that OVA-induced asthmatic mice display the strongest signs of inflammation between 48 h and 72 h after the last antigen challenge (Biffi *et al.*, 2013 ▶). We therefore instilled 6 × 10^6^ barium- and DiD-labelled MΦ resuspended in 30 µl PBS intratracheally (i.t.) 72 h after the last OVA challenge into the lungs of asthmatic and control mice under xylazine-tiletamine-zolazepam anaesthesia.

### 2.4. *In vivo* optical imaging   

Optical imaging was performed by two-dimensional fluorescence reflectance imaging (FRI) using the Optix MX2 system (ART; Montreal, Canada) as previously described (Markus *et al.*, 2014 ▶). For *in vivo* scans, mice were anaesthetized by inhalation with isoflurane (2% isoflurane in 2 l oxygen per min). Before imaging, mice were shaved and chemically depilated over thorax and abdomen to decrease scattering from the fur. Mice were scanned before, directly after and 24 h after MΦ instillation. All data were acquired using a 670 nm excitation laser diode in combination with a 700 Lp emission filter and a 1.0 mm raster. The fluorescence intensity was analysed using the *OptiView-2-02-00* software (ART). The average intensity of the lung area was measured and its relative increase compared with the pre-scan was computed.

### 2.5. Preparation of biological samples for *ex vivo* CT analysis   

**Figure 1:**
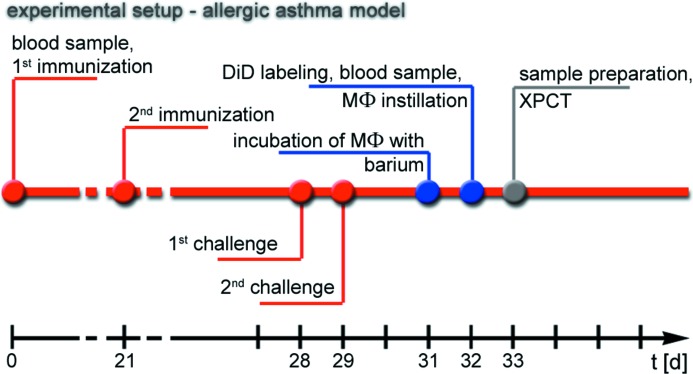
Experimental setup. An OVA allergen-induced experimental allergic airways disease model was used, consisting of two immunization steps (i.p. injection of 10 µg OVA) on days 0 and 21 and two challenging steps (i.n. application of 100 µg OVA) on days 28 and 29. Prior to their instillation, MΦ were labelled with Micropaque and DiD for 24 and 4 h, respectively. On day 32, 6 × 10^6^ barium- and DiD-labelled MΦ were instilled i.t. into asthmatic and control mice. Mice were euthanized on day 33 and prepared for fXPCT analysis.

Mice were sacrificed 24 h after instillation of barium-sulfate-loaded and fluorescent-labelled MΦ (Fig. 1 ▶) using a xylazine-tiletamine-zolazepam overdose. In order to ensure comparability between different samples, all lungs were inflated *in situ* with air, under a constant pressure of 30 cm water column (2.94 kPa) through a series of smaller tubes, which ended in a PE50 polyethylene cannula fixed inside the trachea with a cotton thread. Tracheas were tied up and all samples were kept at room temperature for 2 h, in order to avoid any alterations caused by *rigor mortis*. To avoid air leakage, alterations and movements during the X-ray examination, samples were then embedded in a 1% agarose gel inside 30 ml tubes (Fisher Scientific, USA) and kept for 30 min at 277 K. Samples were moved to the synchrotron beamline 30 min before scanning to allow for temperature adaptation. Four lung samples were prepared for fXPCT analysis: one mouse with OVA-induced asthma and two healthy controls, all injected with MΦ (AA, CN1 and CN2), as well as one healthy mouse without application of MΦ which served as a negative control (Blk).

### 2.6. Synchrotron-radiation-based fXPCT and phase retrieval   

All data sets were acquired at the SYRMEP beamline at the synchrotron light source Elettra (Trieste, Italy) (Brun *et al.*, 2010 ▶; Dreossi *et al.*, 2008 ▶), which is especially designed for medical applications and analysis of biological samples. The beamline was operated at 22 keV at a sample-to-detector distance of 30 cm. The central area of the lung of each sample was scanned by performing two overlapping 360° scans with 1800 projections each and a 2 × 2 binning of the detector elements, thus resulting in a spatial resolution of 9 µm. In order to decouple phase and absorption information in the acquired projection images, we applied a single-distance in-line phase-retrieval algorithm based on the Born equation implemented by Chen *et al.*, which only requires one data set, obtained at a single sample-to-detector distance (Mohammadi *et al.*, 2014 ▶; Chen *et al.*, 2010*b* ▶, 2012 ▶; Taylor, 1981 ▶). In order to apply this class of algorithms, *a priori* knowledge of the δ-to-β ratio within the sample is needed. Within this study we used a δ-to-β ratio of 1950 for standardized lung tissue with a hydrogen, carbon and oxygen ratio: H10 C0.83 O5 (International Commission on Radiological Protection) (Mohammadi *et al.*, 2014 ▶).

### 2.7. Quantification of morphological alterations within the lung and distribution of barium-loaded macrophages   

For a quantitative comparison of the lung samples it is crucial to identify parameters that characterize the alterations in the lung structure without being affected by local in­homogeneities in the manifestation of the asthmatic reaction within the lung. To this end we applied an analysis scheme that was adapted from strategies used for the characterization of trabecular bone structure and other porous materials. Briefly, the data sets were reoriented and resampled to ensure that the analysis is independent of the orientation of the lungs during the scan. Due to the high contrast-to-noise ratio in the phase-retrieved reconstructed data sets, non-overlapping greyscale ranges were assigned to air, lung soft-tissue, bone of the rib cage and to barium used as label for the i.t. instilled MΦ. These image segments were then further analysed and quantified in terms of air, soft-tissue and barium content as well as narrowing of the airways.

### 2.8. Histology   

Lung samples for histology were obtained from a further set of OVA-induced asthmatic and control mice following the schedule and conditions as described in Fig. 1 ▶ (in order to be comparable with the fXPCT analysis). The excised lungs were fixed in 10% buffered formalin and embedded in paraffin, and 3 µm-thick paraffin lung sections containing main stem bronchi were obtained. A periodic acid-Schiff (PAS) staining was performed to assess bronchial wall thickness and mucus production (Fullmer, 1960 ▶). Sections were deparaffinised, rehydrated and stained with periodic acid for 5 min, followed by Schiffs reagent (Merck, Darmstadt, Germany) for 15 min and hematoxylin for 2 min. Slices were washed for 3 min between each of the staining steps. The samples were dehydrated using an ascending alcohol series and Xylol and finally mounted with DePex (Serva, Heidelberg, Germany). An Axioskop 2 (Carl Zeiss Microscopy GmbH, Jena, Germany) microscope in combination with a Leica DC 100 camera (Leica, Switzerland) was used for analysis of the stained sections.

### 2.9. Fluorescence microscopy   

In order to verify the location of the injected DiD and barium-labelled MΦ, fluorescence microscopy of lung tissue sections of asthmatic and control mice was performed, utilizing an Axiovert 200M inverted microscope (Carl Zeiss Microscopy) equipped with a xenon lamp and a high-sensitivity ORCA-AG digital camera (Hamamatsu, Japan), capable of NIRF detection. For this purpose, lungs of asthmatic mice (*n* = 2) and a control mouse, explanted 24 h after MΦ i.t. instillation, were filled with optimal-cutting-temperature (OCT) embedding material (Tissue-Tek; Sakura Finetek, Torrance, CA, USA) and were cryofrozen. Lung cryosections (5 µm) were obtained, fixed with acetone (10 min at 253 K) and washed with tris(hydroxymethyl)-aminomethane (Tris) buffer (pH 7.5). Immunostaining was performed as follows. Autofluorescence was blocked with 0.1 *M* glycin [for 10 min at room temperature (RT)], endogenous biotin and avidin were blocked with Avidin Biotin Blocking Solution and unspecific binding sites for 20 min at RT with SEA BLOCK blocking buffer (both Thermo Scientific) following the manufacturer’s protocol. Slices were then incubated overnight at 277 K with rat monoclonal anti-CD68 antibody (FA-11; Abcam, Cambridge, UK; 3.33 µg ml^−1^) diluted in Antibody Diluent with Background Reducing Components (DAKO, Glostrup, Denmark), followed by two incubation steps of 1 h at RT with biotinylated goat–anti-rat antibody (BioLegend, San Diego, CA, USA; 1:200), and with Streptavidin-AlexaFluor 555 (Molecular Probes, Life Technologies, Carlsbad, CA, USA; 1:400). Finally, slices were mounted with Mowiol (Calibiochem, Merck, Darmstadt, Germany) supplemented with 4′,6-diamidino-2-phenylindole (DAPI) for staining of the nuclei and left overnight at 277 K. Two washing steps with Tris buffer were performed between each step. DiD fluorescence was acquired using a band-pass (BP) 640 ± 15 nm excitation filter, a 660 nm dichroic mirror and a BP 690 ± 25 nm emission filter. The AlexaFluor 555 signals were recorded using a BP 546 ± 6 nm excitation filter, a 580 nm dichroic mirror and a 590 nm long pass emission filter. DAPI fluorescence was acquired using a BP 365 ± 12.5 nm excitation filter, a 395 nm dichroic mirror and a BP 445 ± 25 nm emission filter. In the produced images the DAPI channel was set to blue, AlexaFluor 555 to green and DiD to red. Image generation and processing were performed using the software *AxioVision Rel.4.6* (Carl Zeiss Microscopy GmbH, Jena, Germany) and *ImageJ* (National Institutes of Health, Bethesda, MD, USA) (Collins, 2007 ▶).

### 2.10. High-resolution synchrotron-radiation-based X-ray phase-contrast microCT (HR microCT)   

Lung sections of a third set of OVA-induced asthmatic mice (*n* = 2) and a control mouse following the schedule and conditions as described in Fig. 1 ▶ were also studied using a CT set-up with higher resolution. For this aim the procedure for sample preparation was the following: the trachea was cannulated, then filled and fixed with 4% paraformaldehyde (PFA), and subsequently single lobes of the lung were embedded in a 5% agarose-gel. Finally, 500 µm-thick slices were cut using a Vibratome (Leica VT 1000S; Leica, Switzerland). The slices were placed with a droplet of PBS between two round pieces of polypropylene foil, secured within aluminium rings. These were glued together to create a closed chamber for the slices (Olendrowitz *et al.*, 2012 ▶). These slices were then imaged with a divergent X-ray beam of 17.5 keV at the beamline ID22 Ni at the European Synchrotron Radiation Facility (ESRF) in Grenoble. The beam was focused to less than 100 nm × 100 nm by Kirkpatrick–Baez (KB) mirrors and free propagation phase-contrast images recorded with a FReLoN camera (Analog and Transient Electronic ESRF group) coupled to a scintillator (Weitkamp *et al.*, 1999 ▶). The sample was placed at the maximum possible defocus distance to achieve the highest field of view. Using a ten-fold objective lens behind the scintillator and the magnification of the KB beam an effective pixel size of 430 nm was achieved. Tomographic scans with a series of 1500 images over a full rotation of 360° were recorded for several specimens of asthma and control mice. Phase retrieval was performed using the single-distance Holo-tomo reconstruction algorithm (Cloetens *et al.*, 1999 ▶) implemented at the beamline. Before 3D reconstruction, each projection image was corrected by an image of the empty beam and aligned to the other projections before phase retrieval was performed.

### 2.11. Analysis and statistics   

The 3D rendering of the data sets was performed with *VGStudio Max 2.2* (Volume Graphics, Heidelberg, Germany). For mask generation, *IDL 7.0* (Research Systems; Boulder, CO, USA) and *ImageJ* (National Institutes of Health; Bethesda, MD, USA) were used (Abràmoff *et al.*, 2004 ▶). *Pore3D*, a proprietary software library developed by the SYRMEP group, was applied to analyse the 3D barium distribution as well as to quantify the air and tissue content of the lung within volumes of interest (VOIs) (Brun *et al.*, 2010 ▶). Statistical analysis was performed using *MINITAB* (Minitab Ltd; Coventry, UK) and utilizing a one-way ANOVA test with Tukey 90% simultaneous confidence intervals for the computed parameters (Ryan *et al.*, 2005 ▶; Dunn, 1961 ▶).

## 3. Results   

### 3.1. XPCT in combination with single-distance phase retrieval is a valuable tool for lung imaging   

In order to evaluate the usefulness of phase retrieval in our experiment, we performed two scans of a mouse lung sample in XPCT at sample-to-detector distances of 7 and 30 cm. At the distance of 7 cm only minor edge effects were present and therefore this essentially resembles the absorption-based setup in classical CT, despite the fact that the scan was performed with a monochromatic X-ray source. In contrast, scanning the same sample at a sample-to-detector distance of 30 cm provides sufficient phase contrast. Phase retrieval was applied to the projection images of the second scan before 3D reconstruction with FBP was performed. This procedure matches the same scheme used in this study. As a basis for a quantitative comparison, we calculated the contrast-to-noise ratio (CNR) using the following equation:
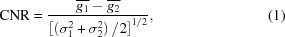
where 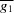 and 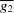 denote the mean grey value of two adjoining tissues and 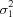 and 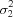 reflect their noise level, measured as squared standard deviation in a region of interest (ROI) (Mohammadi *et al.*, 2014 ▶). In each sample and on five reconstructed slices homogeneously distributed over the whole lung, eight circular two-dimensional ROIs (size ∼0.4 mm^2^) solely containing either air or lung soft-tissue were defined and analysed. We determined a CNR between air and lung-tissue of about 20.0 in the phase-retrieved data set at 30 cm and 1.9 at 7 cm without PhR. In addition, only minor blurring of the phase-retrieved images was found (data not shown). These results demonstrate that the combination of XPCT with single-distance phase retrieval is able to increase the soft-tissue contrast in our samples and setup by a factor of 9.8 when compared with absorption-based monochromatic X-ray imaging. This directly translates into an increased sensitivity that is beneficial for the combined functional and structural CT imaging approach to visualize mouse lung tissue.

### 3.2. Macrophages as specific contrast agent for functional CT   

The barium sulfate uptake efficacy of MΦ was assessed with a bench-top microCT. Imaging of a vial, containing 1 × 10^5^ MΦ loaded with barium sulfate particles and resuspended in 100 µl PBS, showed a 10% increase of the X-ray absorption when compared with a vial containing 1 × 10^5^ unlabelled MΦ as control (data not shown). The WST-1 assay revealed no influences of the phagocytized barium sulfate particles on the metabolic activity of the MΦ (data not shown). Furthermore, no evidence of morphologic alterations was observed in barium-sulfate-loaded MΦ by light microscopy (data not shown). In conclusion, the approach to load MΦ with barium provides sufficient contrast for CT imaging and shows no signs of acute cell toxicity.

### 3.3. *In vivo* optical imaging demonstrates successful intratracheal instillation of MΦ   

**Figure 2:**
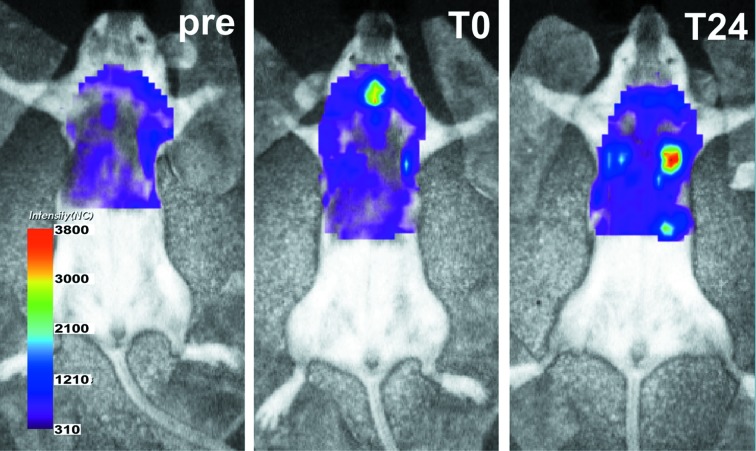
*In vivo* optical imaging results. Fluorescence intensity maps of the same asthmatic mouse used for fXPCT (Fig. 3 ▶) before (pre), directly after (T0) and 24 h after (T24) i.t. instillation of 6 × 10^6^ barium-sulfate-filled and DiD-labelled MΦ are shown. After 24 h, strong fluorescence signals expressed in normalized counts (NC) are visible over the lung area.

*In vivo* NIRF imaging was performed in order to confirm the successful i.t. instillation of the MΦ. For this purpose the MΦ were additionally stained with the NIR cell membrane label DiD. Fig. 2 ▶ shows the *in vivo* optical imaging results for the same asthmatic mouse analysed later by fXPCT. A strong increase in fluorescence intensity was observed over the lung area directly and 24 h after i.t. instillation of DiD- and barium-labelled MΦ when compared with the pre-scan. These results verify the presence of the labelled MΦ in the lung area of the mouse 24 h after i.t. administration.

### 3.4. Processing and quantification of fXPCT data sets   

All lungs were kept *in situ* and scanned at the SYRMEP beamline using a setup that allows for fXPCT. In order to cover the main central area of the lung, two slightly overlapping scans per sample were performed. The original projection data sets were then processed by the single-distance in-line phase-retrieval algorithm (Chen *et al.*, 2010 ▶) to create projections predominately showing the δ part of the complex refractive index. These data sets were later on reconstructed with FBP. In order to analyse the data quantitatively, the following steps were performed: stitching of the two overlapping scans and reorientation of the data sets to allow for comparison between different samples; masking of the lung to restrict the analysis to the lung area; segmentation of the three different components (air, lung soft-tissue and barium); 3D quantification of structural alterations and depiction of the barium concentration and distribution.

#### 3.4.1. Post-processing steps and descriptive comparison of the obtained fXPCT lung data sets   

**Figure 3:**
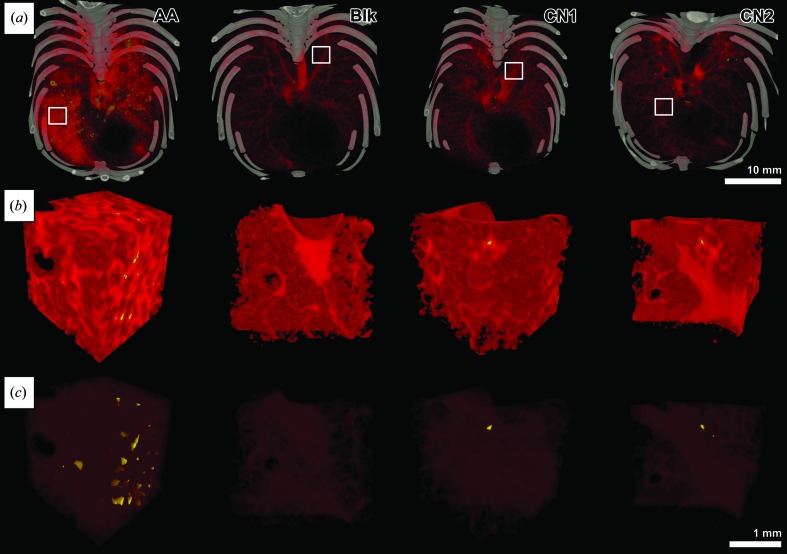
Volume rendering of the fXPCT results and visualization of the MΦ localization. Volume renderings of four 3D reconstructions of phase-retrieved fXPCT data sets are shown. Each column represents one lung sample of an asthmatic mouse (AA), a healthy mouse without instilled MΦ as negative control (Blk), and two healthy controls (CN1, CN2). Bone is displayed in grey, soft-tissue in red and barium in yellow (air is not depicted). (*a*) Illustration of the entire field of view: more barium and an increased soft-tissue density is shown in AA. (*b*) VOIs (their origin is indicated by white squares in row 1). (*c*) The same VOIs are shown but with higher transparency to allow visualization of the sites where barium-filled MΦ cluster in more detail. Note that in the VOIs of the AA lung a higher barium content is visible compared with the results obtained in lungs of the negative control Blk and the healthy controls CN1 and CN2. Additionally, the VOIs of the AA sample show a higher soft-tissue content in comparison with the VOIs of all controls, reflecting a reduced pulmonary volume and increased soft-tissue content in the asthmatic lung tissue.

The automatic quantification of anatomical alterations is based on the splitting of the data sets into rectangular VOIs. Thus, to ensure that all data sets are present in the same orientation, the reconstructed data of the two scanning steps were registered, combined and re-orientated. In consideration of the memory limitations in the post-processing algorithms, data sets were re-sampled down to an isotropic resolution of 14.4 µm. A volume-rendering representation of the final data sets is shown in Fig. 3(*a*) ▶ and VOIs in Figs. 3(*b*) and 3(*c*) ▶. To visualize the various tissues as well as the i.t. instilled barium-sulfate-loaded MΦ, pseudo-colours were assigned to different greyscale ranges. As a result, lung soft-tissue is displayed in red, bones in grey and highly dense areas related to the barium-containing MΦ appear yellow. In order to maintain the 3D visibility of the inner lung structure the air was set to transparent. Given that barium causes a greater phase shift than lung soft-tissue and the healthy blank (Blk) contains no barium, the upper limit of the grey value range representing the lung soft-tissue in Blk was chosen as a valuable threshold to detect the barium-loaded MΦ.

**Figure 4:**
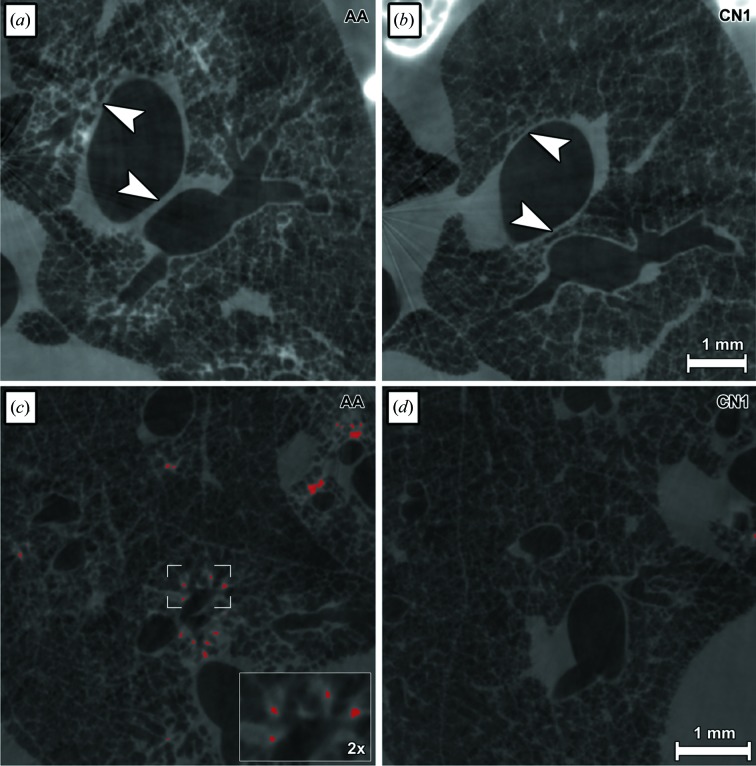
Manifestation of the structural alterations in asthmatic lung tissue and localization of the barium-sulfate-labelled macrophages. Detailed views of single slices of the PhR reconstruction are shown in (*a*) and (*c*) for the asthmatic sample (AA) and in (*b*) and (*d*) for one control (CN1). Indicated by white arrow heads in (*a*) and contrary to the same locations marked in (*b*), thickening of the bronchial walls as well as increased soft-tissue content in the smaller airways can be seen in the asthmatic lung sample. In (*c*) and (*d*) areas within the grey value range assigned to barium are marked in red, indicating the location of the instilled macrophages. Note that the barium-containing macrophages can be localized within the soft tissue, showing the ‘red dots’ completely surrounded by tissue.

The segmented images clearly show an increased soft-tissue content in sample AA in comparison with the healthy controls [Figs. 3(*a*) and 3(*b*) ▶]. Additionally, the VOI of AA (Fig. 3*b* ▶) displays a reduced porosity, which illustrates the airway obstruction characteristic for asthma. The same effect of increased soft-tissue content and airway obstruction can to a certain extent also be seen in the planar slices in Figs. 4(*a*) and 4(*b*) ▶ (white arrow heads). Furthermore, Fig. 3 ▶ demonstrates that the barium-sulfate-loaded MΦ seem to be distributed in cluster-like structures throughout the asthmatic lung, which is also demonstrated in planar reconstructed slices in Figs. 4(*c*) and 4(*d*) ▶. High-contrast regions related to barium-sulfate-loaded MΦ are also present in the lungs of the two healthy controls CN1 and CN2; however, at a much lower quantity (yellow spots, Fig. 3*b* ▶). Note that MΦ derived signals within the lung in AA appear to be surrounded by lung soft-tissue and therefore seem to be originating from areas around the bronchial walls and not from the lumen of the airways (Fig. 3*b* ▶). The same location of MΦ was also found in two-dimensional slices as shown in Fig. 4(*c*) ▶. In the same slices areas can be found that are solely composed of soft tissue that is characterized by a lower contrast than the marked spots assigned to barium. Therefore, it can be excluded that the detected spots are caused by partial-volume effects. Due to the fact that no other strong absorbing material is present in the lung, these high-dense spots most likely represent the instilled barium-labelled MΦ. Therefore, the detected localization of the MΦ within the bronchial walls may indicate an active migration of the instilled MΦ from the airspace into the tissue.

#### 3.4.2. Development of an image-processing scheme for automatic 3D quantification of morphological alterations   

Since lung tissue has a tree-like structure of airways with different capillarity, the depiction of the VOIs are of crucial importance for a VOI-based analysis scheme. On the other hand, the instilled barium-labelled MΦ produce a weak signal and might have an inhomogeneous distribution in such a way that they could be easily missed in an approach focused on the entire lung. In order to perform a quantitative comparison, entire lungs were therefore subdivided into three sets of 2 mm-thick bands in the horizontal, vertical and frontal direction. In the so-generated 32 VOIs per sample, two types of parameters were analysed: (i) volume ratio (Vol. Ratio), which defines the volume fraction of a tissue or material of interest compared with the total volume of a VOI, and (ii) structural thickness (St. Th.) of either the airways or the surrounding tissue. Structural thickness is calculated by analysing the maximal size of spheres which can be inscribed in the structure as proposed by Hilde­brand & Ruegsegger (1997 ▶) and implemented in the software *Pore3D* (Brun *et al.*, 2010 ▶). These two parameters are directly related to airway wall thickening and airway obstruction and therefore reflect structural changes characteristic for asthmatic lung tissue.

#### 3.4.3. Quantification of morphological changes in the lung   

In addition to visual inspection of the rendered fXPCT data sets, quantification of structural changes was a further aim of the study. For this purpose the average and standard variation of the parameters Vol. Ratio and St. Th. were calculated in each sample and in each of the horizontal, vertical and trans-vertical sets of VOIs, respectively (Table 1 ▶). We found an approximately 17% reduced air content as well as a 61% increase in the soft-tissue Vol. Ratio in the AA sample compared with all controls (Table 1 ▶). In addition, airway obstruction as a hallmark of asthma is clearly reflected in the results of the St. Th. measurements showing a 32% St. Th. reduction of the mean airway thickness in AA compared with all healthy animals and, *vice versa*, a 48% higher St. Th. in the lung soft-tissue (Table 1 ▶).

In order to prove whether the results obtained in the individual VOIs of each data set are significantly different in between the samples, we used a one-way ANOVA test with Tukey 90% simultaneous confidence intervals to test the difference of the mean values of the analysed parameters (Vol. Ratio of air and soft-tissue and St. Th. of airways and tissue) (Ryan *et al.*, 2005 ▶). The comparison of AA and Blk revealed reliable differences for all analysed parameters. Even the minor differences of these parameters between Blk, CN1 and CN2 were found to be statistically significant, demonstrating the sensitivity of our fXPCT approach. Therefore, based on the chosen parameters (air and soft-tissue volume ratio and mean airway and soft-tissue thickness), the asthmatic and the three control lung samples can be successfully distinguished, indicating that this parameter set can be used to preclinically monitor structural changes in asthmatic lungs.

In addition, the histogram of the δ distribution within the samples, which was normalized to the total amount of analysed voxels for each mouse, showed a more dominant interval in AA (grey values 45–55, data not shown). This result suggests that during the course of asthma not only structural but also changes in the composition of the lung tissue occur, which may be the result of the presence of oedema, an increased mucus production or alterations in the collagen fibres within the lung.

#### 3.4.4. Quantitative analysis of the barium concentration within the lungs   

As shown in Fig. 3 ▶ (in yellow), barium-filled MΦ can be detected in AA and to a lower extent in CN1 and CN2. Following the same scheme used to quantify the soft-tissue content, but applied to the barium segment of the data set, we found a Vol. Ratio of about 0.1% for barium in the AA lung (Table 1 ▶). The barium concentration in the two healthy controls CN1 and CN2 was below 1‰ 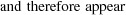 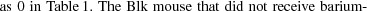 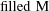
Φ (Blk) was used to set up the threshold and consequently shows no barium content within the whole lung. The Tukey intervals for barium content (data not shown) are all positive and therefore confirm that the Vol. Ratio of the barium content is significantly larger in the AA lung sample than in the Blk and CN mice.

In summary, bronchial wall thickening and airway obstruction in asthmatic mouse lung tissue can be visualized in fXPCT data sets [Figs. 3 ▶, 4(*a*) and 4(*b*) ▶]. Instilled MΦ appear within clusters around the bronchial walls [Figs. 3 ▶, 4(*c*) and 4(*d*) ▶]. With our approach we can also quantify these alterations using automatically generated 3D morphologic parameters. These parameters, *i.e.* Vol. Ratio of air, barium and lung soft-tissue and St. Th. of air and lung soft-tissue, showed significant differences between AA and healthy controls in accordance with the known pathological features in lungs of an asthma mouse model.

### 3.5. Fluorescence microscopy and HR microCT confirm the accumulation of MΦ in asthmatic lung tissue   

In order to confirm the accumulation and to further analyse the location of the instilled barium-filled and DiD-labelled MΦ within the lung observed by fXPCT, we performed fluorescence microscopy and HR microCT on lung sections. Since the preparation process and the analysis at the SYRMEP beamline resulted in the deterioration of the samples and excluded histological analysis, fluorescence microscopy and HR microCT were performed with lungs of a different cohort of mice, but strictly following the same protocol for asthma induction and instillation of MΦ (Fig. 1 ▶), including the use of the same batch of OVA.

**Figure 5:**
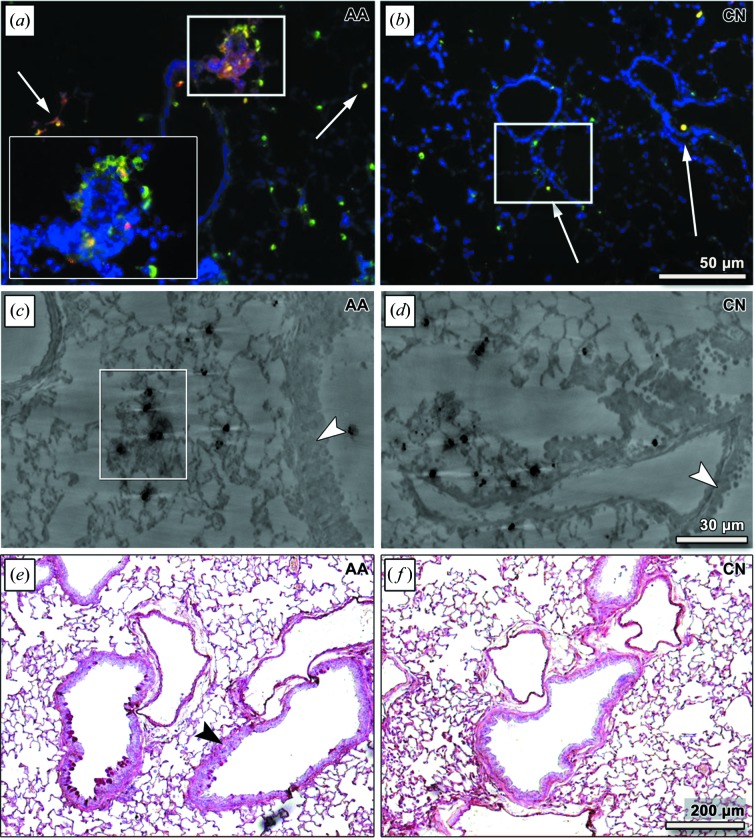
Validation of the fXPCT results by fluorescence microscopy, HR microCT and histology. (*a*, *b*) Fluorescence microscopy of lung sections of (*a*) an asthmatic (AA) and (*b*) a control mouse (CN). Nuclei were stained with DAPI (blue) and MΦ were immuno-stained with an anti-CD68 antibody (green). DiD-labelled MΦ are shown in red, whereas instilled MΦ appear yellow due to the double staining with CD68 and DiD. Endogenous MΦ are shown in green and can be seen in alveoli of both AA and CN lungs, but seem to form clusters in AA (*a* and *b*). Instilled DiD-labelled MΦ are also visible in both AA and CN lung samples (white arrows). In contrast to the CN, the AA sample shows high cellular density areas (white rectangle and detailed view), where several CD68 positive cells and clusters of instilled MΦ can be observed. Unlike the AA sample, the CN does not show these areas, which may explain the increased soft-tissue ratio and the airway obstruction found in the fXPCT results. (*c*, *d*) Representative cross sections of HR microCT for AA and CN vibratome lung section. An increased wall thickness (white arrow head) for AA (*c*) and the location of the labelled MΦ (black dots) can be clearly depicted. MΦ can be seen in both CN and AA, (*c*) shows an area with higher cell density and accumulation of MΦ (white rectangle). (*e* and *f*) PAS-stained lung sections of an asthmatic (AA) and a control mouse (CN). Increased wall thickness in asthmatic lung tissue (black arrow head) is depicted. Red dots are exclusively visible in asthmatic lungs and depict mucus production.

Representative results of the fluorescence microscopy performed on cryosections of a lung of an asthmatic mouse (AA) and a control lung (CN) are shown in Figs. 5(*a*) and 5(*b*) ▶. Instilled barium- and DiD-labelled MΦ are shown in red, DAPI stained nuclei appear blue and an anti-CD68 antibody (Ab) which binds to both the applied and endogenous MΦ is shown in green. As a result, the instilled barium- and DiD-labelled MΦ appear yellow in the merged image. We observed DiD-labelled MΦ in the small alveoli [Figs. 5(*a*) and 5(*b*) ▶, white arrows] in both the CN and the AA lung samples. As already seen in the fXPCT analysis, more instilled MΦ are present in the AA lung. Moreover, only in the asthmatic tissue were we able to detect an accumulation of instilled MΦ within lung areas of high cellular density [Fig. 5(*a*) ▶, detailed view]. These clusters of cells were found throughout the lung and in close proximity to the bronchi. Additionally, a higher number of endogenous MΦ (green) was found in the asthmatic lung [Fig. 5(*a*) ▶, green staining].

HR microCT examination clearly verified the presence of the barium-filled MΦ inside the lung (dark spots), as shown in Figs. 5(*c*) and 5(*d*) ▶, for two lung sections from an asthmatic and a control mouse, respectively. In both asthmatic and healthy lungs, barium-filled MΦ were detected throughout the lung tissues. However, in contrast to the healthy sample, the asthmatic sample additionally displayed areas around the bronchi with an increased accumulation of MΦ (white rectangle). Furthermore, thickening of the bronchial walls was observed in the asthmatic tissue [Fig. 5(*c*), white arrow head] compared with the healthy control [Fig. 5(*d*) ▶], reflecting the presence of the disease.

Figs. 5(*e*) and 5(*f*) ▶ show PAS-stained histological slices from lungs of an OVA-induced asthma mouse (AA) and a healthy control (CN). Red dots indicate that mucus production is solely present in the asthmatic lung (Fig. 5*e* ▶). Furthermore, airway wall thickening can be observed (black arrow head) in the AA slide only. These findings represent typical characteristics of asthma and confirm the presence of an acute asthma reaction in these mice. In addition, ELISA analysis of the sera revealed increased IgG1 titre for all asthma mice (data not shown), verifying a successful immunization.

## 4. Discussion   

This study presents a novel functional in-line free propagation X-ray phase-contrast CT imaging approach (fXPCT) that enables the depiction of both structural features of lung tissue and the accumulation of barium-labelled MΦ in the lungs of mice after intratracheal instillation. We show that fXPCT can be applied for CT-based immune cell tracking studies. Furthermore, this method allows the quantification of structural changes in whole asthmatic lungs *in situ* by measuring parameters like the volume ratios of air, soft-tissue and the mean airway and soft-tissue thickness, thereby assessing hallmarks of asthma such as bronchial wall thickening and airway obstruction.

By applying a single-distance phase-retrieval algorithm (Paganin *et al.*, 2002 ▶) to decouple phase from absorption information, we raised the CNR of low-absorbing unstained lung soft-tissue by a factor of ten when compared with classical FBP (Mohammadi *et al.*, 2014 ▶; Chen *et al.*, 2010 ▶). It has to be emphasized that this factor is strongly related to the experimental setup used, including the sample-to-detector distance, the type of samples, the pixel size of the camera system, the quality of the X-ray beam, the phase-retrieval and reconstruction algorithms, and does therefore not represent a general rule for comparing phase-retrieved with non-phase-retrieved CT reconstructions (Donnelly *et al.*, 2003 ▶). In a different setup even a factor of up to 200 was reported by Beltran *et al.* (2011 ▶). Another way to improve the detectability of contrast agents would be *K*-edge imaging (Cormode *et al.*, 2010 ▶), in spite of the increase in sensitivity in *K*-edge imaging in combination with a non-energy-resolved detector requiring two scans with at least one using a photon energy higher than optimal for soft-tissue contrast.

Single-distance phase retrieval uses, as the name implies, only one projection to calculate the δ-distribution of the refractive index. Therefore, it simplifies the experimental setup and minimizes the acquisition time. This is of particular advantage for unfixed biological samples as used in this study, as it reduces the influence of alterations that occur within the samples over time. However, single-distance phase retrieval is in principle based on the assumption that the studied sample is a ‘homogeneous’ object, meaning it only contains one known δ-to-β ratio (Chen *et al.*, 2011 ▶; Gureyev *et al.*, 2009 ▶). Since this is not the case for our biological samples, a δ-to-β ratio optimized for lung tissue was used. Lung is only composed of low-*Z* materials (*Z* < 10) for which Wu *et al.* (2005 ▶) showed already that single-distance phase-retrieval algorithms can be applied. Nevertheless, the generated reconstructions are not valid for the analysis of dense structures like bony components (Beltran *et al.*, 2011 ▶). If the skeleton is of interest, an additional phase-retrieval step in combination with a different δ-to-β ratio should be applied as shown by Beltran *et al.* (2011 ▶). Here we consider a low concentration of barium-sulfate-loaded MΦ (less than 0.2%) only a minor disturbance of the assumed ‘homogeneity’ within the lung although barium has a *Z* value of 56. The calculated ten-fold increase in CNR is based on the comparison with a scan that uses the same setup, but with a minimized sample-to-detector distance, generating projection images with predominately absorption-based contrast. Therefore, the reconstructed absorption-based data set was acquired with quasi-monochromatic X-rays known to generate higher-quality data than conventional microCTs with the same spatial resolution (Sera *et al.*, 2005 ▶). Thus, the gain in contrast with single-distance phase retrieval is even more prominent when compared with conventional bench-top microCT scans. This increase in contrast directly leads to an increased sensitivity, which in our case did not only enable the depiction but also the quantification of parameters of structural changes in the entire asthmatic lung in 3D, namely the volume ratios of air, soft-tissue and the mean airway thickness, and was the prerequisite for the detection of the labelled MΦ.

In contrast to other studies that analyse mouse lung structure at a micrometre scale (Yong *et al.*, 2009 ▶; Sera *et al.*, 2005 ▶; Kitchen *et al.*, 2005 ▶), we kept the lung *in situ* by filling the airways with air at a constant pressure. We believe that in this way the shape of the lung, which is critically dependent on the pressure and the boundaries given by the ribcage, can be compared with an *in vivo* situation. In previous studies using the same asthma mouse model and sample preparation scheme, we found that the overall lung volume (soft-tissue and air content) was greater in asthmatic than in the healthy mice. That means that the asthmatic lungs can be more inflated at the same pressure pointing to a change in the elasticity of the lung tissue. This finding may be reflected in the altered δ-value distribution of the soft-tissue in the asthmatic sample and supports the understanding that in asthma, in addition to inflammation, changes in the composition of lung tissue occur, that lead to a loss in elastic recoil. This loss of elasticity was already observed in patients (Gelb & Zamel, 2002 ▶; Gelb *et al.*, 2002 ▶) and confirmed our previous studies.

With this study we present a novel set of parameters [volume ratio (Vol. Ratio) and structural thickness (St. Th.) of airways and soft-tissue] and show their potential use in describing differences between asthmatic and healthy lung tissue. By applying fXPCT we determined a 17% reduced air content, a 32% reduced mean airway thickness and a 61% increase in the soft-tissue content in the asthmatic lung tissue compared with controls, demonstrating that in contrast to histology or conventional CT our approach can precisely measure structural alterations and illustrate them in 3D. The inflammatory response in asthma eventually leads to bronchial obstruction, caused by structural abnormalities such as hypertrophy of airway smooth muscle, sub-epithelial fibrosis, goblet cell hyperplasia, and proliferation of airway blood vessels and nerves. Since routinely most of these morphological changes of airway remodelling are visualized histologically in lung sections in both humans and mouse models (Al Heialy *et al.*, 2011 ▶; Blacquière *et al.*, 2010 ▶; Leong & Huston, 2001 ▶; Epstein, 2004 ▶), the quantification of these changes has been difficult to date. While there are limited approaches by conventional CT and MRI, these techniques are restricted by a low resolution. In particular, minor alterations of the lung tissue in asthma cannot be visualized with the limited spatial resolution of clinical CT scanners. Therefore, up to now the diagnosis of asthma in CT is based on lung densitometry measuring air trapping caused by disturbed ventilation of the lung (Washko *et al.*, 2012 ▶). Our approach provides novel parameters which may used in both preclinical asthma models and clinical practice to classify and monitor asthma of different severity and/or to access the influence of an asthma therapy on airway remodelling.

In addition to anatomical information, the increased sensitivity of our fXPCT technique in comparison with conventional microCT allows for the depiction of the distribution of i.t. instilled barium-sulfate-loaded immortalized alveolar macrophages as ‘physiological’ contrast agent within the 3D lung structure. This, to our knowledge, represents the first approach of using barium-sulfate-loaded macrophages as contrast agents for CT-based cell tracking studies in an experimental allergic airways disease model. So far the use of synchrotron-radiation-based CT for cell tracking has only been reported in a few applications such as imaging small clusters of tumour cells *ex vivo* loaded with gold nanoparticles (Astolfo *et al.*, 2013 ▶). The visualization of pancreatic islet cells encapsulated in barium microcapsules is another example for CT-based cell tracking (Arifin *et al.*, 2012 ▶). We found a higher barium content in the asthmatic lung sample, which is most likely due to a more clustered distribution of the instilled macrophages in cell-dense areas around the bronchi, a finding which needs further investigation by high-resolution tomography. This result is supported by our validation experiments with fluorescence microscopy and HR microCT, all techniques that share a higher spatial resolution than the used fXPCT approach. In our experiment in asthmatic lung tissue, 24 h after instillation, MΦ were predominately found in areas with an increased cell density. While the implications of these findings for the pathomechanism of asthma and other lung disease models require further investigation, our results demonstrate the benefit of our imaging technique for 3D localization of cells in specific tissue regions. As the MΦ used in this study are derived from an immortalized cell line (Mbawuike & Herscowitz, 1989 ▶), no conclusions towards a similar behaviour of endogenous MΦ can be made.

A current drawback of our method for future clinical application is the lack of clinical CT systems that provide phase contrast. This limitation may soon be overcome by the implementation of novel technologies such as liquid-metal jet anode systems, miniaturized synchrotrons or grating-interferometer-based phase-contrast imaging utilizing classical X-ray sources (Pfeiffer *et al.*, 2006 ▶; Tompkins *et al.*, 1998 ▶; van Heekeren *et al.*, 2011 ▶). All of these techniques perform well under laboratory conditions but struggle with different technical problems, which limit their use in the clinic so far. In particular, the high radiation dose for grating-based phase-contrast devices hamper their clinical application at the moment (Raupach & Flohr, 2011 ▶). However, our quantification scheme for analysis of lung structure alterations can be directly translated to any other CT or anatomical lung imaging technique, providing a spatial resolution high enough to resolve the lung substructure.

Our approach presents a proof-of-principle study for fXPCT imaging that specifically visualizes and quantifies morphological differences and airway remodelling of the mouse lung and will support the preclinical validation of newly developed targeted diagnostics and drug delivery strategies for lung diseases. Furthermore, our novel imaging approach provides a solid system for cell tracking studies of immune cells to investigate the role that macrophages might play in the development and progression of lung diseases such as asthma.

## Figures and Tables

**Table 1:** Mean values standard deviation of the considered parameters The pooled standard deviations for the Vol.Ratio (%) parameter were 0.04 for barium, 4.95 for alveoli and 4.94 for tissue. For the St.Th. (m) parameter the pooled standard deviation were 68 for alveoli and 11 for soft tissue.

	Sample	Barium	Air	Soft tissue
Vol.Ratio (%)	AA	0.10 0.07	64.62 6.78	35.28 6.75
	Blk	0.00 0.00	78.32 3.43	21.68 3.43
	CN1	0.00 0.00	74.28 4.75	25.72 4.75
	CN2	0.00 0.00	81.85 4.21	18.15 4.21
St.Th. (m)	AA		188 39	83 15
	Blk		254 53	54 9
	CN1		256 83	60 9
	CN2		315 87	53 7
